# Analysis of Epidemiological and Economic Impact of Foot-and-Mouth Disease Outbreaks in Four District Areas in Thailand

**DOI:** 10.3389/fvets.2022.904630

**Published:** 2022-06-21

**Authors:** Thanicha Chanchaidechachai, Helmut Saatkamp, Chaidate Inchaisri, Henk Hogeveen

**Affiliations:** ^1^Business Economics Group, Wageningen University and Research, Wageningen, Netherlands; ^2^Research Unit of Data Innovation for Livestock, Department of Veterinary Medicine, Faculty of Veterinary Science, Chulalongkorn University, Bangkok, Thailand

**Keywords:** foot-and-mouth disease, economic, epidemiology, farm losses, morbidity, mortality

## Abstract

Foot and mouth disease (FMD) is one of the most important infectious animal diseases impacting livestock production in Thailand. Despite a national vaccination program, FMD outbreaks are reported every year. We studied the epidemiological impacts of FMD outbreaks in four districts of Thailand between 2015 and 2016. Epidemiological data were collected from 193 FMD-affected dairy farms, 55 FMD-affected beef farms, and 25 FMD-affected pig farms. A significant difference in morbidity rates were observed between the dairy farms in the different areas, which could be explained by the differences in FMD outbreak management in each area. The morbidity rates in dairy and beef cattle also significantly differed between each animal age category, with the lowest morbidity rate observed in calves. Remarkably, vaccination was not significantly associated with the morbidity rate. In addition, the economic impact of FMD was calculated for 60 dairy farms in Muak Lek district. The economic losses were determined as the sum of milk production loss, mortality loss, additional labor costs, and veterinary service and medical costs, which averaged 56 USD per animal on the farm (ranging from 2 to 377 USD). Milk loss had the largest economic impact, although it varied substantially between farms. The farm size and outbreak duration were significantly associated with the total economic losses per farm. These results affirm the substantial epidemiological and economic impact of FMD on farms in Thailand, emphasizing the importance of FMD control.

## Introduction

Livestock production is an important economic activity in Thailand, contributing 12.6% of the country's gross agricultural production (1). The economic growth of Thailand and the increasing income per capita has led to a higher domestic demand for animal-derived food, while the demand from international markets such as China also drives the export of live animals and animal products (2). As demand increases, livestock farms in Thailand have grown and adopted more intensive farming practices (3); however, this growth may intensify the risk and impacts of livestock diseases.

Foot and mouth disease (FMD) is a very important livestock disease in Thailand (4). This highly infectious viral disease affects cloven-hoofed animals, including pigs and large and small ruminants (5). Due to its high transmissibility, FMD is endemic in many parts of the world, including Africa, the Middle East, some parts of South America, and Asia, including Thailand (6).

The economic impact of FMD outbreaks in FMD-free countries is explicit. Enormous direct losses are related to eradication and the indirect losses from closed export markets. By contrast, the economic impact of FMD in FMD-endemic countries is less clear, especially compared with other high-mortality animal diseases (7). The economic losses caused by FMD in its endemic areas have typically been estimated based on direct production losses, such as milk loss, mortality loss, and draft power loss (8–10). Knight-Jones and Rushton proposed a framework for estimating the economic impact of FMD in endemic areas, defining the following factors: (1) visible losses due to milk loss and mortality loss; (2) invisible losses due to changes in herd structure and fertility problems; (3) additional costs due to control measures and treatment; and (4) revenue forgone due to loss of market access or the use of a suboptimal breed. The economic impact of FMD in the endemic regions of the world is estimated to be 6.5 to 21 billion USD per year (11).

FMD is an endemic disease in Thailand, with regular reports of outbreaks all over the country. Between 2007 and 2017, 968 FMD outbreaks were reported in Thailand, especially between 2015 and 2016, when outbreak reports peaked at 183 (4). The FMD prevention strategies in Thailand include routine vaccination programs in ruminants two times per year, or up to three times per year in areas with regular FMD outbreaks (12). The government supports vaccination by providing free locally produced trivalent FMD vaccines (O, A, and Asia1 strains). Although the vaccination of ruminants is compulsory supported by government, the vaccination of pigs is voluntary depended on the farmers. If an FMD outbreak occurs, the local authorities could respond by announcing the outbreak zone, enforcing emergency vaccination procedures, and applying animal movement restrictions within the outbreak zone (13). However, the outbreak detection mostly depends on the passive surveillance from farmer report, and the intensity of outbreak management are at the local authority's discretion. In order to make good decisions, it is important for regional authorities to have insight into the epidemiological and economic consequences of an outbreak (14). Moreover, for farmers, an understanding of the consequences of FMD outbreaks is important when making decisions regarding the prevention and control of FMD.

Even though Thailand has been regularly affected by FMD, only a few epidemiological and economic assessment studies were conducted. The previous studies mostly focused on the area level rather than the farm level (12, 13). The knowledge gap of FMD impacts at the farm level in Thailand exists. This study therefore aims to explore the morbidity and mortality of FMD on dairy, beef, and pig farms using data from four FMD-affected districts, in addition to assessing the economic impact of FMD in dairy farms in Thailand.

## Materials and Methods

### Data Collection

**Figure 1:**
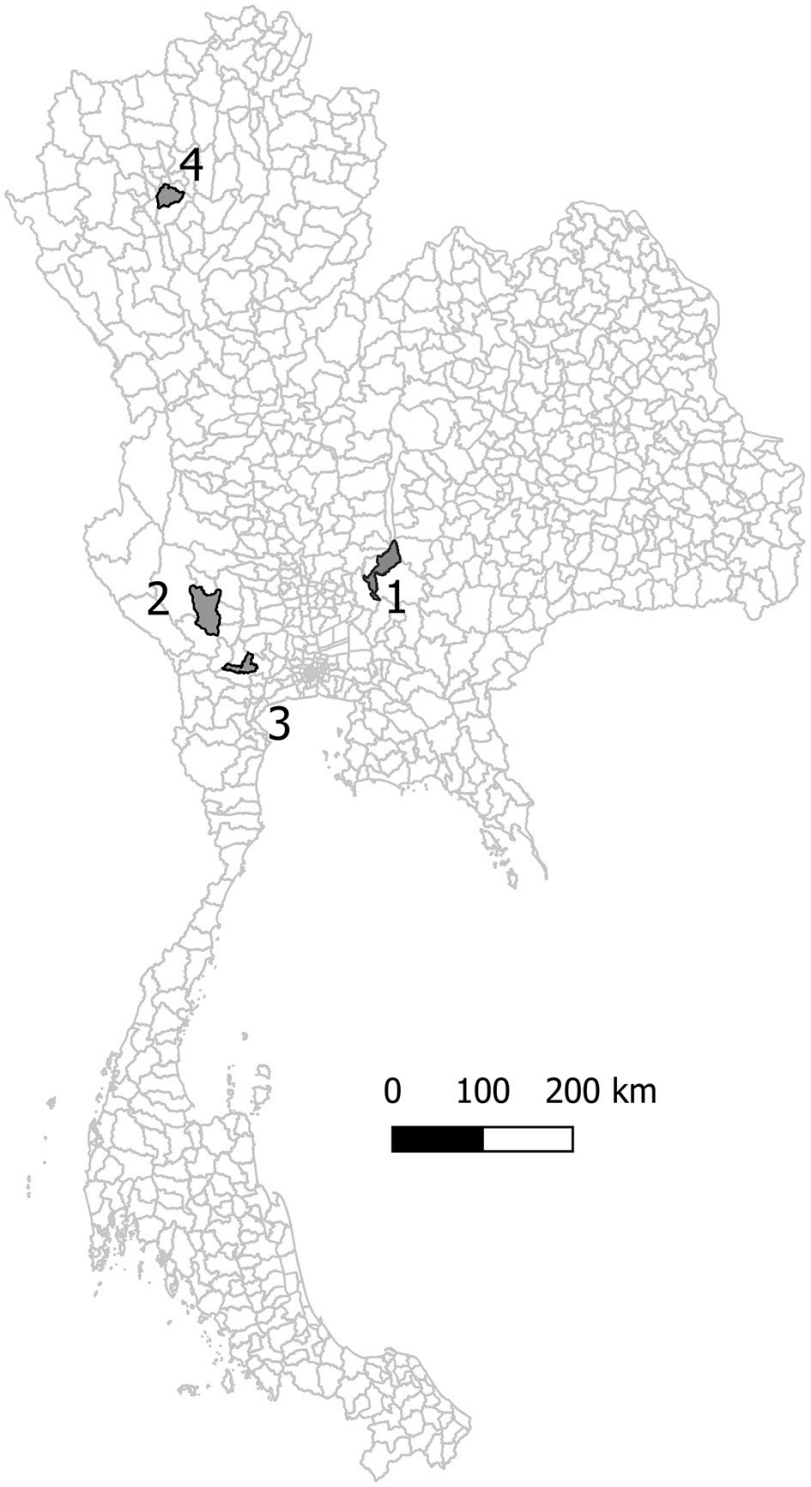
The study areas: (1) Muak Lek district, (2) Boploy district, (3) Banpong districts and (4) Mueang Lamphun district.

Epidemiological data for FMD outbreaks between 2015 and 2016 were collected from four study districts in Thailand. These four study areas were selected from a consultation of Thailand's Department of Livestock Development (DLD) based on the criteria of a high density of livestock, differing structures of livestock farming, and the occurrence of FMD outbreaks between 2015 and 2016. The selected study areas were Muak Lek district, Boploy district, Banpong district, and Mueang Lamphun district (Figure 1). These four districts are in different regions of Thailand and have distinct farm characteristics. Muak Lek district has the highest number of dairy cattle in Thailand, Boploy district is predominated by beef cattle farms, and Banpong and Mueang Lamphun districts have a mix of dairy cattle farms, beef cattle farms, and pig farms (15).

FMD-affected farms in the study areas were identified based on clinical signs by the local veterinary officers. For each FMD-affected farm, a farmer interview was conducted by local veterinary officers and research staff of the Veterinary Epidemiology and Animal Health Economics group of Chulalongkorn University, Bangkok, Thailand, using questionnaires in Thai (Supplementary Material S1). The staff were briefed on the questionnaires before the interviews started. The interview data consisted of the farmer's name, the geographical coordinates of the farm, the livestock species, the number of livestock, the outbreak start date, the outbreak end date, the vaccination practices, and the number of animals sick or dead with FMD symptoms in each animal category (defined by age and production status). Dairy cattle were categorized into calves (≤ 6 months old), heifers, and cows. Beef cattle were categorized into calves (≤ 6 months old) and adult cattle. Pigs were categorized into young pigs (suckling pigs and weaners), fattening pigs, and sows. FMD-affected animals were defined as animals that were sick or dead and showed clinical signs of FMD, i.e., lameness; drooling saliva; or vesicles on the udder, hoof, or tongue.

In addition, economic data were collected in Muak Lek district. Sixty FMD-affected dairy farmers from the epidemiological study were randomly selected to be interviewed about the economic impact of the FMD outbreak using an additional questionnaire (Supplementary Material S1). The economic data consisted of the reduction in milk production, milk price, outbreak duration, additional labor time, and the number of veterinarian visits. The interviews were conducted by the research staff. Additional economic data, including the prices of cattle, veterinary services, and medication, were estimated based on the expertise of local veterinarians and dairy cooperative staff, who were interviewed after the FMD outbreak (Table 1). The monetary data were collected in Thai baht prices and were converted to US dollars (USD) using the conversion rate of 1 USD to 35 Thai baht according to the average exchange rate in 2016 (16).

**Table 1:** Input parameters for economic estimation in dairy cattle farms.

**Parameters (unit)**	**Notation**	**Value or calculation**	**Sources**
Calf price (USD)	*Pcalf*	85.7	Experts
Heifer price (USD)	*Pheifer*	1,571.4	Experts
Wage rate (USD/day)	*W*	8.6	Authors
Veterinary service fee (USD/animalvisit)	*vet*	5.7	Experts
Average medicine cost (USD/ animal)	*med*	28.6	Experts

*1 USD is approximated to 35 Thai Baht according to the average exchange rate in 2016 (16)*.

### Epidemiological Analysis

The overall cumulative incidence was calculated by dividing the total number of animals with clinical FMD signs by the total number of animals on the farm the day before the outbreak. This calculation was repeated for the cumulative incidence in each animal category, in which the number of sick animals with clinical FMD signs in each category was divided by the number of animals in the same category. The cumulative mortality was calculated in the same manner, using the number of dead animals that showed clinical FMD symptoms as the numerator.

A generalized linear model was used to study the effect of related factors on the count data of morbidity. The model was fitted separately for each animal species. The dependent variable was the number of sick animals with clinical FMD signs in each animal category for each farm. The log number of animals at risk was used as an offset to interpret the outcome as a morbidity rate. The independent variables were the study area, animal category, and FMD vaccination practice (≤ 1, 2, and ≥3 vaccinations per year). Due to the high turnover rate of animals in pig farms, the vaccination practices were based on the vaccination program in the sows. In addition, the study area was not included as an independent variable in the pig farm analysis since the number of infected pig farms in Mueang Lamphun district was very small (*n* = 2).

Due to the overdispersion of the data (the dispersion parameters in dairy cow = 4.8, beef cattle = 1.4 and pig = 55.5), a Poisson regression model could not be used, but generalized Poisson and negative binomial regression models were considered suitable alternatives (17, 18). The full model was fitted using both generalized Poisson and negative binomial regression and tested for zero-inflation using DHARMa package (19). If both models can handle the zero-inflation, the model with the lower Akaike information criterion (AIC) was chosen. After that, the stepwise backward selection process was conducted to choose the final model by excluding independent variables from the full nested model until the model with the lowest AIC was identified. The details on the model selection are shown in Supplementary Material S2.

The mortality data were analyzed as a binary outcome (the absence/presence of dead animals with clinical FMD symptoms). A Chi-squared test was used to determine the statistical significance of the association between the presence of dead animals with FMD and the animal category or the vaccination practices. If the expected counts in the cell of the contingency table were below five, Fisher's exact test was used instead (18).

### Estimation of Economic Impact

From the 60 dairy farms in Muak Lek district, the economic impact of FMD at each farm, *i*, was calculated as the sum of milk production loss (*Lmilk*_*i*_), mortality loss (*Lmort*_*i*_), additional labor cost (*Llabor*_*i*_), and veterinary service and medical costs (*Ltreat*_*i*_).

The milk production loss for each farm, *i*, was estimated using the milk loss in animals that showed acute clinical FMD signs (9):


(1) $$\begin{array}{r} Lmilk_{i}=NSlact_{i}*ML_{i}*D_{i}*Pmilk \end{array}$$

where *NSlact*_*i*_ is the number of FMD-affected lactating cows on farm *i*, *ML*_*i*_ is the average reduction in milk yield in kilogram (kg) per FMD-affected cow per day on farm *i*, *D*_*i*_ is the outbreak duration in days on farm *i*, and *Pmilk* is the milk price.

The mortality losses in calves and heifers were based on the market price of the animals. The mortality losses in lactating cows and dry cows were calculated based on the cost of a replacement heifer adjusted with a depreciation factor reflecting the parity of the dead cows (19). According to the interview data, most farms did not sell the carcasses; therefore, potential revenues from selling the carcasses were not included in the calculation.


(2) $$\begin{array}{l} Lmort_{i}=\left(NDcalf_{i}*Pcalf\right)+\left(NDheifer_{i}*Pheifer\right)\\ +\;\left[\left(NDlact_{i}+NDdry_{i}\right)*Pheifer*adj_{dairy}\right] \end{array}$$

where *NDcalf*_*i*_ is the number of dead calves on farm *i*, *Pcalf*is the market price of a calf, *NDheifer*_*i*_ is the number of dead heifers on farm *i*, *Pheifer* is the market price of a heifer, *NDlac*_*i*_ is the number of dead lactating cows on farm *i*, *NDdry*_*i*_ is the number of dead dry cows on farm *i*, and *adj*_*dairy*_ is the adjustment factor for cow depreciation. Because data about the parity of the dead cows were not available, we set the adjustment factors as a median of 0.5.

During the outbreak, the farmers and household members spent extra time nursing the sick animals and managing the farms. The extra labor hours were converted to a monetary value for additional labor costs (20).


(3) $$\begin{array}{r} Llabor_{i}=HL_{i}*D_{i}*W/8 \end{array}$$

where *HL*_*i*_ is the extra labor hours per day during the outbreak on farm *i*, *D*_*i*_ is the outbreak duration on farm *i*, and *W* is the wage rate per day.

The veterinary service and medical costs were calculated as


(4) $$\begin{array}{r} Ltreat_{i}=\left(NS_{i}*vet*visit_{i}\right)+\;\left(NS_{i}*med\right) \end{array}$$

where *NS*_*i*_ is the number of sick animals on farm *i*, *vet* is the veterinary service cost per animal per visit, *visit*_*i*_ is the number of veterinarian visits on farm *i* during the outbreak, and *med* is the average medical cost per sick animal.

After calculating the economic losses, a linear regression model was fitted to identify the factors that were associated with the economic losses. The dependent variable was the total economic losses per farm. The independent variables were farm size, outbreak duration, and vaccination practice (≤ 1, 2, and ≥3 vaccinations per year). The final model was chosen by the lowest AIC. All statistical analyses were performed using R version 3.6 (21).

## Results

### Epidemiological Analysis

Epidemiological data were collected from 193 FMD-affected dairy farms, 55 FMD-affected beef farms, and 25 FMD-affected pig farms in the four districts (Table 2). More than 75% of the farms vaccinated their animals at least twice per year, except for the beef cattle farms in Banpong district, of which only 21% of farms vaccinated their animals at least twice per year.

**Table 2:** Number, species, herd size and vaccination practices of foot-and-mouth disease affected farms in the four studied districts with foot-and-mouth disease outbreaks.

**Areas**	**Species**	**Number of FMD affected farms**	**Herd size** **Mean (min, median, max)**	**Vaccination practices** ***N*** **(%)**		
				**≤1 per year**	**2 per year**	**≥3 per year**
Muak Lek district	Dairy	110	50 (7, 42, 160)	14 (12.7%)	20 (18.2%)	76 (69.1%)
Boploy district	Beef	26	81 (20, 56, 300)	5 (19.2%)	17 (65.4%)	4 (15.4%)
Banpong district	Dairy	41	37 (8, 28, 118)	1 (2.4%)	14 (34.2%)	26 (63.4%)
	Beef	29	32 (2, 20, 129)	23 (79.3%)	5 (17.2%)	1 (3.5%)
	Pig	23	7,202 (115, 4,570, 30,000)	5 (21.7%)	5 (21.7%)	13 (56.6%)
Mueang Lamphun district	Dairy	42	45 (5, 42, 130)	2 (4.8%)	6 (14.3%)	34 (80.9%)
	Pig	2	67 (55, 67, 78)	0	2	0

The cumulative incidence and cumulative mortality for each animal category are given as percentages in Table 3. The cumulative incidence varied substantially between the districts and animal categories. For the dairy cattle farms, the highest overall cumulative incidence and cumulative mortality were found in Muak Lek district, with cows having the highest cumulative incidence and calves having the lowest. For beef cattle farms, the overall cumulative incidence in Banpong district was higher than in Boploy district, with adult cattle facing higher cumulative incidence than the calves. For pig farms, the overall cumulative incidence in Banpong district was higher than the overall cumulative incidence in Mueang Lamphun district; fattening pigs had the highest cumulative incidence.

**Table 3:** Average foot-and-mouth disease cumulative incidence and cumulative mortality of each animal category in farms by areas.

**Species**	**Cumulative incidence (%)**				**Cumulative mortality (%)**			
	**Muak Lek**	**Boploy**	**Banpong**	**Mueang Lamphun**	**Muak Lek**	**Boploy**	**Banpong**	**Mueang Lamphun**
**Dairy**								
Calf	19.6	**-**	12.1	11.5	2.1	**-**	0	0
Heifer	29.4	**-**	34.3	24.1	0.9	**-**	0.3	0
Cow	49.6	**-**	31.4	17.8	0.7	**-**	0.6	0
Overall	41.1	**-**	33.0	16.5	1.0	**-**	0.5	0
**Beef**								
Calf	**-**	6.6	11.5	**-**	**-**	0	0	**-**
Adult	**-**	34.2	48.8	**-**	**-**	0	0	**-**
Overall	**-**	31.5	43.4	**-**	**-**	0	0	**-**
**Pig**								
Young	**-**	**-**	13.0	0	**-**	**-**	8.3	0
Sow	**-**	**-**	24.2	0	**-**	**-**	0.8	0
Fattener	**-**	**-**	35.1	12.6	**-**	**-**	4.9	0
Overall	**-**	**-**	33.4	4.7	**-**	**-**	5.6	0

“*-” Means no FMD outbreak report in this animal species in this district*.

The morbidity rate ratio, confidence interval, and significance value from the best fit models are shown in Table 4. For the dairy farm, the morbidity rates in Banpong district and Muak Lek district were 1.22 and 1.97 times higher, respectively, than the morbidity rate in Mueang Lamphun district. In addition, the morbidity rates in the heifer and cow groups were 1.86 and 2.43 times higher than the calf group, respectively. For the beef cattle farms, we did not find a significant difference in morbidity rates between outbreak areas; however, we did find that the morbidity rate in the adult cattle group was 4.09 times higher than in the calf group. For the pig farms, we did not see any statistically significant associations between morbidity rates and independent variables.

**Table 4:** Morbidity rate ratio, confidence interval and significance value from the best-fit models.

**Species**	**Variables**	**Rate ratio**	**95%CI**	***P-*value**
Dairy cattle	Areas			
	Mueang Lamphun	Ref.		
	Banpong	1.22	0.90–1.64	0.197
	Muak Lek	1.97	1.57–2.49	<0.001
	Animal categories			
	Calf	Ref.	
	Heifer	1.86	1.34–2.60	<0.001
	Cow	2.43	1.80–3.28	<0.001
Beef cattle	Animal categories			
	Calf	Ref.		
	Adult	4.09	2.25–7.40	<0.001

Mortality from FMD was not observed in dairy farms in Mueang Lamphun district, and the overall cumulative mortality in the other three districts were <1%. The mortality in beef cattle farms was not calculated because only one dead calf was reported on a farm in Boploy district. No mortality was reported in the two FMD-affected pig farms in Mueang Lamphun districts. Fisher's exact tests showed no significant associations between the presence of animals that died with FMD and the related factors of animal category or vaccination practices.

### Economic Impact

The economic losses were calculated for 60 FMD-affected dairy cattle farms in Muak Lek district (Table 5). The average total economic losses per animal were 56 USD, with a range of 2–377 USD per animal. The mean daily milk loss per animal varied substantially, with a range of 0.7–17.4 kg per cow per day. Consequently, the average milk losses per farm were 1,063 USD, with a range of 6–14,688 USD per farm. The average mortality losses per farm were 532 USD, with a range of 0–6,286 USD per farm. The highest economic losses per animal were due to milk production loss, which was, on average, 19 USD per animal, followed by mortality loss which on average, 18 USD per animal.

**Table 5:** Foot-and-mouth disease economic losses from outbreak between 2015 and 2016 in dairy cattle farms in Muak Lek district (*n* = 60).

**Economic variables**	**Descriptive statistics**			
	**Mean**	**Min**	**Median**	**Max**
Daily milk loss due to FMD (kg/cow/day)	5.0	0.7	4.8	17.4
Milk price (USD/kg)	0.48	0.41	0.49	0.51
Duration of outbreak (day)	21	6	28	45
Extra labor during the outbreak (hours/day)	3.5	0	4	10
Milk production loss (USD/farm)	1,063	6	726	14,688
Milk production loss (USD/animal)	19	0.1	11	128
Mortality loss (USD/farm)	532	0	0	6,286
Mortality loss (USD/animal)	18	0	0	314
Additional labor (USD/farm)	86	0	60	300
Additional labor (USD/animal)	2	0	1	19
Veterinary service and medicine (USD/farm)	661	29	600	2,514
Veterinary service and medical costs (USD/animal)	14	0.7	14	34
Total economic losses (USD/farm)	2,454	79	1,678	17,720
Total economic losses (USD/animal)	56	2	36	377

*1 USD is approximated to 35 Thai Baht according to the average exchange rate in 2016 (16)*.

Linear regression was used to study the association between the total economic losses per farm and the putative influencing factors, including farm size, vaccination practice, and outbreak duration. Due to the big range of total economic losses per farm, we used the natural-logarithm transformation of total economic losses per farm as a dependent variable. Farm size and outbreak duration were found to significantly influence the log-transformed total economic losses of the farms (Table 6). For every one animal increase in the farm, the FMD total economic losses per farm increase 4.5% (*p* value < 0.001), and for every one day increase in the duration of the outbreak, the FMD total economic losses per farm increase 1.4% (*p* value = 0.017). The overall regression model was significant [F (2.57) = 12.7, *p* value < 0.001] with an adjusted *R*^2^ of 0.28.

**Table 6:** The significant variables related to the log-transformed of total economic losses per farm from foot-and-mouth disease in dairy farm (*n* = 60).

**Variables**	**Coefficient**	**95%CI**	***P-*value**
Intercept	5.65	4.96–6.35	<0.001
Farm size	0.014	0.003–0.026	<0.001
Outbreak duration	0.04	0.02–0.07	0.017

## Discussion

This study is the first to explore the epidemiological and economic impacts of FMD on farms in Thailand, and one of the few studies on the economic impact of this disease in endemic areas. We found that FMD cumulative incidence and cumulative mortality on the farms varied largely between the four outbreak areas and the animal age categories. The difference in morbidity between areas might be due to differences in outbreak management between these regions. According to the Animal Epidemics Act (of Thailand) B.E. 2558, during a FMD outbreak, veterinary offices and local governments have the authority to implement movement restrictions and emergency vaccination in an outbreak zone. As a consequence, the level of outbreak response depends on the local authority's judgement, facilities, and resources in each area.

The morbidity rate in calves was significantly lower than in adult animals for both dairy cattle and beef cattle. In Thailand, it is recommended that calves receive a first vaccination dose at 4 to 6 months of age, with a booster dose 1 month later; however, the booster dose was frequently missed by farmers. Young animals were therefore expected to be the most vulnerable group in the population due to inadequate immunity (22), which was contradicted by our findings. This incongruity might be explained by calf management practices; most farmers keep calves in separate housing, away from adult animals, meaning they are less likely to be in contact with other animals (23).

Mortality was exceedingly low in beef cattle farms, with only one dead animal reported. The low mortality on beef cattle farms might be explained by breed, as FMD clinical signs are milder in the Asian native cattle breeds commonly raised in Thailand (24); only a minor percentage of beef cattle in Thailand are crossbred exotic breeds (25). Beef cattle in Thailand are therefore expected to be more resistant to FMD, leading to low mortality. Due to the mild clinical signs and low mortality, the beef cattle farmers, especially small free-grazing beef cattle farmers, might have low incentives to vaccinate their animals (12), as reflected in the low vaccination percentage in beef cattle in Banpong district. The FMD vaccination campaign should be promoted among beef cattle farmers to ensure sufficient vaccination coverage.

Surprisingly, we did not find a significant association between the epidemiological impact of FMD outbreaks and vaccination practices. This finding might be explained by the poor duration of the vaccine-induced immunity. A study on antibody titers in 403 dairy cows in Lamphaya Klang subdistrict, Thailand, showed that only 60% of the cattle had an antibody titer above the protection level 3 months after vaccination; moreover, this percentage decreased to 30% by 5 months after vaccination (unpublished data from FMD Thailand project PRP 5905021280). Another explanation might be the low specificity of the vaccine for the circulating strain. In 2016, a new FMD strain, O/ME-SA/Ind-2001d, was reported in 11 provinces of Thailand, including the provinces of our study areas (26). The previous vaccine before the outbreak in 2016 might not be good compatible with the new introduced strain, hence it did not provide full protection.

The economic assessment of the FMD-affected dairy farms indicated that the reduced milk yield during a FMD outbreak was the most significant economic loss; however, we found a large variation in milk losses between the farms and consequently a large variation in the economic impact of FMD. Several factors can influence the amount of milk loss, e.g., the amount of milk production before the outbreak, the severity of the clinical signs, and the measures taken after the outbreak. We note that the milk loss in this study was calculated based on information from cows with signs of acute clinical FMD, with the implicit assumption that milk yield returned to normal after recovery; however, some studies have indicated that this might not be the case. One study in Pakistan showed that milk yields were significantly lower 2 months after the onset of clinical FMD (10), while a study in Kenya reported an increased incidence of mastitis in the first month of the outbreak, affecting milk yields in the long term (27). The calculation of milk loss only for cows with acute clinical signs most probably resulted in an underestimation of the actual milk loss. Moreover, more losses could occur if farmers cannot sell milk due to the outbreak control policy or market ban; for example, during the 2016 FMD outbreak in Chiang Mai province, Thailand, the milk collection center banned milk sales from FMD-affected farms for 30 days as a FMD control measure. The average monetary losses amounted to 3,355 USD per farm (28), which is three times higher than the milk losses without a trade ban estimated in the present study.

The second largest economic loss was mortality loss. The average mortality loss amounted to 532 USD per farm, which is half as large as the average milk loss. Because the mortality varied substantially between farms, the total losses could be very high for farms with many dead animals. The costs of additional labor, veterinary services, and other medical costs were much lower than the milk production and mortality losses. Investment in treatment and intensive management during the outbreak to reduce clinical signs and prevent mortality could therefore benefit farms. Moreover, we found that the farm size and outbreak duration were significantly related to the total economic losses per farm.

The high cumulative incidence and cumulative mortality of FMD in pig farms, especially in the Banpong district, suggests that the economic impact of FMD outbreaks in pig farms could be enormous, particularly as this disease affects pigs in all age categories (29). A previous study showed that the economic impact is exceptionally high in large-scale intensive pig farms (30). Follow-up research should therefore explore the economic consequences of FMD on pig production. Moreover, pigs could be amplifier hosts that spread disease to other neighboring farms, particularly because the amount of airborne virus dispersed by infected pigs is about 60-fold higher than ruminants (31). Consequently, FMD infection in pig farms could intensify the impact of outbreaks in the area, making disease control particularly important for the pig farms. For beef cattle, even though FMD causes very low mortality, it could be a big hurdle when exporting beef cattle to other countries, leading to indirect economic effects. Controlling FMD in beef cattle is therefore also important to preserve the export market.

Some potential limitations of this study should be considered. First, the case definition using the clinical signs could underestimate the number of cases, especially in the vaccinated animals. FMD vaccine could suppress the clinical signs and lead to subclinical infection (24). Moreover, several diseases such as swine vesicular disease, vesicular stomatitis, and bovine viral diarrhea, show similar clinical signs as FMD, which might cause misdiagnosis (32). Second, we only assessed the direct economic losses. Therefore, indirect losses, such as a change in herd structure, fertility problems, and long-term effects on production (11), could not be included in the economic assessment due to a lack of data. The same holds for revenues forgone, such as denied market access. As a consequence, the results of this study should be interpreted as a conservative estimation of the actual economic losses, which, in reality, may have been much higher.

This study affirms the prominence of the economic impact of FMD on dairy farms. The average yearly profit of dairy farms in Thailand amounts to 150 USD per animal (33), while the average economic impact of FMD is 56 USD per animal, or one third of the annual profit. The insights of this study justify the benefit of FMD control and provide the incentive for farmers to support the FMD-control program. Moreover, Thailand is participating in a campaign to prevent, control, and eradicate FMD in South-East Asia and China (SEACFMD), and has set the goal to be FMD free with vaccination (34). Perry et al. estimated the economic viability of FMD control in Thailand, revealing a benefit-cost ratio for achieving a FMD-free status of 1.72 without the potential benefits of exports (35), providing the Thai government with a high incentive for FMD control. The epidemiological and economic data from this study provide information that will help the authorities to develop and evaluate the FMD control program in Thailand.

This study is one of few studies that reported epidemiological and economic data at the farm level in Thailand and provides insight into the factors related to the impact of FMD. It is the first study to report FMD mortality and morbidity in pig farms in Thailand. In this study, we only showed the economic impacts on dairy farms. The economic impact assessment on beef cattle and pig farms should be conducted in further study to gain insight into FMD economic loss in the endemic areas. Our results demonstrate that FMD has a prominent epidemiological and economic impact on livestock farms in Thailand, highlighting the need for, and benefit of, disease control.

## Data Availability Statement

The datasets presented in this study are openly available in Supplementary Materials.

## Author Contributions

TC, HS, CI, and HH designed study, analyzed data, and edited manuscript. CI and TC collected the data. TC drafted and statistical analyses. All authors contributed to the article and approved the submitted version.

## Funding

The data in this work were provided by the FMD project (PRP 5905021280) funded by the Agricultural Research Development Agency (ARDA). The Anandamahidol foundation funded the Ph.D. scholarship of TC.

## Conflict of Interest

The authors declare that the research was conducted in the absence of any commercial or financial relationships that could be construed as a potential conflict of interest.

## Publisher's Note

All claims expressed in this article are solely those of the authors and do not necessarily represent those of their affiliated organizations, or those of the publisher, the editors and the reviewers. Any product that may be evaluated in this article, or claim that may be made by its manufacturer, is not guaranteed or endorsed by the publisher.
