# Supplementary material for: Analysis of Epidemiological and Economic Impact of Foot-and-Mouth Disease Outbreaks in Four District Areas in Thailand
Source: Front Vet Sci. 2022 Jun 21;9:904630. doi: 10.3389/fvets.2022.904630 (PMC9253695; doi:10.3389/fvets.2022.904630)
Supplement: Supplementary file 5 [file Data_Sheet_1.docx]

**Supplementary Material S1.**

**Questionnaire for investigating FMD outbreak in farms**

- **For the dairy farm**

Date______________ record ID______________

Farmer name______________ Address______________ Telephone______________

1. Population in the farm on the day before the outbreak

- The number of milking cow ___________

- The number of dry cow ___________

- The number of heifer ___________

- The number of calf ≤ 6 month old ___________

- The number of bull ___________

2. Did your farm affected by FMD outbreak between 2015 and 2016? □ Yes □ No

If yes, please answer the following questions:

3. The outbreak duration

- Which date did the first animals show FMD clinical signs? _______________

- Which date did the last animals stop showing FMD clinical signs? _______________

4. Mortality and morbidity

- The number of sick milking cow ___________ The number of dead milking cow ___________

- The number of sick dry cow ___________ The number of dead dry cow___________

- The number of sick heifer ___________ The number of dead heifer ___________

- The number of sick calf ___________ The number of dead calf ___________

- The number of sick bull ___________ The number of dead bull ___________

5. Vaccination practices

- How often did you do the FMD vaccination in the farm? _________ times/year

- Which type of vaccination did you use? __________________

- Who was the vaccinator? __________________

- **For the beef farm**

Date______________ record ID______________

Farmer name______________ Address______________ Telephone______________

1. Population in the farm

- The number of adult cow ___________

- The number of calf ≤ 6 month old ___________

2. Did your farm affected by FMD outbreak between 2015 and 2016? □ Yes □ No

If yes, please answer the following questions:

3. The outbreak duration

- Which date did the first animals show FMD clinical signs? _______________

- Which date did the last animals stop showing FMD clinical signs? _______________

4. Mortality and morbidity

- The number of sick adult cow ___________ The number of dead adult cow ___________

- The number of sick calf ___________ The number of dead calf___________

5. Vaccination practices

- How often did you do the FMD vaccination in the farm? _________ times/year

- Which type of vaccination did you use? __________________

- Who was the vaccinator? __________________

- **For the pig farm**

Date______________ record ID______________

Farmer name______________ Address______________ Telephone______________

1. Population in the farm

- The number of fattening pig ___________

- The number of sow___________

- The number of young pig (piglet and weaner) ___________

2. Did your farm affected by FMD outbreak between 2015 and 2016? □ Yes □ No

If yes, please answer the following questions:

3. The outbreak duration

- Which date did the first animals show FMD clinical signs? _______________

- Which date did the last animals stop showing FMD clinical signs? _______________

4. Mortality and morbidity

- The number of sick fattening pig ___________ The number of dead fattening pig ___________

- The number of sick sow ___________ The number of dead sow___________

- The number of sick young pig ___________ The number of dead young pig ___________

5. Vaccination practices

- How often did you do the FMD vaccination in the farm? _________ times/year

- Which type of vaccination did you use? __________________

- Who was the vaccinator? __________________

**Questionnaire for estimating the FMD economic impact on dairy farms**

Date______________ record ID______________

Farmer name______________ Telephone______________

- The impact on milk production

1. number of milking cow ______________ head

2. Average milk yield per cow per day before the outbreak ______________ kg/ day

3. Average milk yield per cow per day after the outbreak ______________ kg/ day

4. Outbreak duration ______________ day

5. The milk price per kg ______________ Thai Baht/ kg

6. Did you stop selling milk during the outbreak? □ Yes □ No

6.1 If yes, how many day did you stop selling milk? _____________ day

- The impact on other costs

7. Did you use more disinfectant during the outbreak? □ Yes □ No

7.1 If yes, how much was the disinfectant cost? ___________ Thai Baht

8. Did you use the veterinary service for treating the sick animals during the outbreak?

□ Yes □ No

8.1 If yes, how many time the veterinarian visit the farms during the outbreak? ______

9. Did you need to work longer or hire extra labour during the outbreak? □ Yes □ No

9.1 If yeas, how many hour per day did you work longer or hire the extra labour?

_______ hour/day
