# Supplementary material for: Analysis of Epidemiological and Economic Impact of Foot-and-Mouth Disease Outbreaks in Four District Areas in Thailand
Source: Front Vet Sci. 2022 Jun 21;9:904630. doi: 10.3389/fvets.2022.904630 (PMC9253695; doi:10.3389/fvets.2022.904630)
Supplement: Supplementary file 6 [file Data_Sheet_2.docx]

**Supplementary S2. Model selection**

# **Epidemiological models**

The model was fitted separately for each animal species. The dependent variable was the number of sick animals with clinical FMD signs in each animal category for each farm. The log number of animals at risk was used as an offset to interpret the outcome as a morbidity rate. The independent variables were the study area, animal category, and FMD vaccination practice (≤ 1, 2, and ≥ 3 vaccinations per year).

The dispersion parameters showed the over-dispersion of the data (the dispersion parameters in dairy cow = 4.8, beef cattle = 1.4 and pig = 55.5). Therefore, we could not use Poisson model. We fitted the generalized Poisson and negative binomial regression models with all variables. Then we tested for zero-inflation. If both models can handle zero-inflation, we choose the model that has lower AIC. After that, we used backwards stepwise based on AIC to choose the variables that were included in the final model. The AIC for each model is shown in Table S2.1.

**Table S2.1** The Akaike information criterion (AIC) of the models for each species. The dependent variable was the number of sick animals with clinical FMD signs in each animal category for each farm. The log number of animals at risk was used as an offset.

| **Species** | **Model** | **Description** | **AIC** | **df** |
| --- | --- | --- | --- | --- |
| Dairy cattle | 1  2*  3  4  5 | Generalized Poission with intercept + areas + animal categories + vaccination  Generalized Poisson with intercept + areas + animal categories  Generalized Poisson with intercept + areas  Generalized Poisson with intercept + animal categories  Generalized Poisson with intercept | 2496.0  2493.3  2528.5  2530.8  2558.3 | 8  6  4  4  2 |
| Beef cattle | 1  2  3  4*  5 | Negative binomial with intercept + areas + animal categories + vaccination  Negative binomial with intercept + areas + animal categories  Negative binomial with intercept + areas  Negative binomial with intercept + animal categories  Negative binomial with intercept | 452.8  451.7  470.6  452.6  471.2 | 6  4  3  3  2 |
| Pig | 1  2  3  4* | Negative binomial with intercept + animal categories + vaccination  Negative binomial with intercept + animal categories  Negative binomial with intercept + vaccination  Negative binomial with intercept | 627.3  630.0  628.2  628.7 | 6  4  4  2 |

* the best model based on AIC. If the AIC did not differ than 2, the model with lower degree of freedom was chosen.

# **Economic models in dairy farms**

The linear regression models were performed to test the effect of putative risk factors on the sub-components of the total economic losses. The farm size, outbreak duration and vaccination practice were assigned as independent variables, and the natural log-transformed of milk losses, mortality losses, additional labour costs and veterinary and medicine costs per farm were assigned as the dependent variables. The results show in Table S2.2. We did not see the significant effect of vaccination practices on any sub-components of the total economic losses.

**Table S2.2.** The linear regression models of the log-transformed total milk losses, mortality losses, labour costs and veterinary costs per farm from foot-and-mouth disease on dairy farms (n=60)

| **Dependent variables** | **Independent variable** | **coefficient** | **95%CI** | ***P* value** |
| --- | --- | --- | --- | --- |
| log (total milk losses per farm) | Intercept  Outbreak duration  Farm size  Vaccination practices  - 1 per year  - 2 per year  - ≥ 3 per year | 3.78  0.08  0.018  Ref.  -0.19  -0.27 | 2.82 – 4.74  0.05 – 0.11  0.005 – 0.032  -1.06 – 0.67  -1.03 – 0.49 | < 0.001  <0.001  0.009  0.65  0.49 |
| log (mortality losses per farm) | Intercept  Outbreak duration  Farm size  Vaccination practices  - 1 per year  - 2 per year  - ≥ 3 per year | 1.22  0.009  0.006  Ref.  -0.29  0.46 | -1.57 – 4.02  -0.078 – 0.097  -0.033 – 0.046  -2.82 – 2.23  -1.76 – 2.68 | 0.38  0.84  0.75  0.82  0.68 |
| log (additional labour cost per farm) | Intercept  Outbreak duration  Farm size  Vaccination practices  - 1 per year  - 2 per year  - ≥ 3 per year | 1.20  0.08  0.008  Ref.  1.08  0.21 | -0.08 – 2.49  0.04 – 0.12  -0.010 – 0.026  -0.09 – 2.24  -0.81 – 1.23 | 0.07  <0.001  0.39  0.07  0.68 |
| log (veterinary service and medicine costs  per farm) | Intercept  Outbreak duration  Farm size  Vaccination practices  - 1 per year  - 2 per year  - ≥ 3 per year | 5.03  0.016  0.02  Ref.  -0.37  -0.25 | 4.35 – 5.72  -0.005 – 0.038  0.01 – 0.03  -0.98 – 0.25  -0.79 – 0.30 | <0.001  0.13  <0.001  0.24  0.37 |

|  |  |  |  |
| --- | --- | --- | --- |
